# Effect of Pressure-Controlled Versus Volume-Controlled Ventilation on Gastric Insufflation During i-gel Use in Adults: A Randomised Controlled Trial

**DOI:** 10.3390/medicina62091657

**Published:** 2026-08-29

**Authors:** Jiwon Lee, Hyoung Woo Chang, Min-Soo Kim, Hae Dong Kim, Joung Goo Cho, Hyun Joo Kim

**Affiliations:** 1 Department of Anesthesiology and Pain Medicine, Anesthesia and Pain Research Institute, Yonsei University College of Medicine, Gangnam Severance Hospital, Seoul 06273, Republic of Korea; belief705@yuhs.ac; 2 Department of Thoracic and Cardiovascular Surgery, Seoul National University Bundang Hospital, Seongnam-si 13620, Gyeonggi-do, Republic of Korea; chang.hyoungwoo@gmail.com; 3 Department of Anesthesiology and Pain Medicine, Anesthesia and Pain Research Institute, Yonsei University College of Medicine, Severance Hospital, Seoul 03722, Republic of Korea; kmsviola@yuhs.ac; 4 Department of Anesthesiology and Pain Medicine, National Health Insurance Service Ilsan Hospital, Goyang-si 10444, Gyeonggi-do, Republic of Korea; hdkim0306@yuhs.ac (H.D.K.); anaper@nhimc.or.kr (J.G.C.)

**Keywords:** i-gel, supraglottic airway, gastric insufflation, gastric ultrasonography, antral cross-sectional area, pressure-controlled ventilation, volume-controlled ventilation

## Abstract

*Background and Objectives:* Gastric insufflation may occur during positive-pressure ventilation through a supraglottic airway when airway pressure exceeds the seal pressure. Because pressure-controlled ventilation (PCV) lowers peak inspiratory pressure compared with volume-controlled ventilation (VCV), it is widely assumed to reduce gastric insufflation—an assumption that remains untested in adults. We therefore compared these two ventilation modes during i-gel ventilation. *Materials and Methods:* In this single-centre, assessor-blinded randomised controlled trial, 68 adults undergoing elective breast surgery under general anaesthesia with an i-gel and neuromuscular blockade, without a gastric drain tube, were allocated 1:1 to PCV or VCV (tidal volume 8 mL/kg of actual body weight). The primary outcome was the postoperative gastric antral cross-sectional area measured by ultrasonography, an indirect measure of gastric gas. The pre-specified primary analysis was a Student’s *t*-test. Secondary outcomes included peak inspiratory pressure and oropharyngeal leak pressure; gastric insufflation, defined post hoc as a >30% increase in antral area, was assessed as an exploratory outcome. *Results:* All 68 participants (34 per group) were analysed. The postoperative antral cross-sectional area did not differ between groups (mean difference, PCV − VCV, −23.7 mm^2^; 95% CI −73.9 to 26.4; *p* = 0.347; *p* = 0.568 after adjustment for baseline); the confidence interval excluded the 100 mm^2^ difference the trial was powered to detect. Gastric insufflation occurred in 2/34 (VCV) versus 3/34 (PCV) (*p* = 1.000). Peak inspiratory pressure and expiratory tidal volume were lower with PCV (all *p* ≤ 0.002), whereas oropharyngeal leak pressure was similar; in every patient, peak pressure remained below the leak pressure. No adverse events occurred. *Conclusions:* In adults ventilated through an i-gel at the low airway pressures this device produces, PCV lowered peak inspiratory pressure but did not reduce the postoperative antral cross-sectional area or the incidence of gastric insufflation compared with VCV. Because delivered tidal volume was also lower with PCV, the peak pressure difference is reported descriptively. Within this low-pressure range, and in this selected population, the ventilation mode did not detectably affect gastric insufflation; the trial does not establish equivalence between the modes or extend to higher airway pressures or obesity.

## 1. Introduction

The i-gel (Intersurgical Ltd., Wokingham, UK) is a second-generation supraglottic airway with a non-inflatable thermoplastic cuff and an integral gastric channel. It is inserted rapidly and atraumatically, achieves a high oropharyngeal leak pressure, and has a first-attempt success rate of more than 90% in routine practice [1]. For these reasons, supraglottic airways are increasingly used for positive-pressure ventilation during general anaesthesia.

Unlike a tracheal tube, however, a supraglottic airway does not isolate the trachea from the oesophagus. When the airway pressure generated during positive-pressure ventilation exceeds the seal pressure of the device, gas can be diverted into the oesophagus and stomach, producing gastric insufflation [2]. Even modest gastric insufflation may raise intragastric pressure, splint the diaphragm, reduce pulmonary compliance, and increase the risk of regurgitation and pulmonary aspiration [3]. This concern is greatest when no gastric drain tube is in place to vent the stomach. Bedside ultrasonography of the gastric antrum now allows this risk to be assessed quantitatively, and the antral cross-sectional area provides a reproducible, quantitative measure of gastric antral content [4,5].

Because the peak inspiratory pressure required to deliver a given tidal volume is lower with pressure-controlled ventilation (PCV) than with volume-controlled ventilation (VCV) [6,7], PCV is widely assumed to be “gentler” on the stomach and is often preferred when ventilating through a supraglottic airway. Yet a lower peak pressure is only advantageous if it actually reduces gastric insufflation, and this assumption has rarely been tested directly.

The available evidence is limited and, in places, conflicting. In children, pressure-controlled ventilation with a laryngeal mask achieved the target end-tidal carbon dioxide more reliably than volume-controlled ventilation [7]; in contrast, a randomised trial with the i-gel found that, although PCV produced a lower peak inspiratory pressure, the occurrence of gastric insufflation was similar between modes [8]. That trial, however, was conducted in children aged 6–84 months, used peak inspiratory pressure as its primary endpoint, and detected gastric insufflation only qualitatively, and its authors noted that the results could not be extrapolated to adults. In adults, gastric insufflation with supraglottic airways has been examined mainly by comparing different devices [9], rather than by comparing ventilation modes. Thus, whether the lower airway pressure of PCV translates into less gastric insufflation in adults—or whether insufflation is instead governed by the absolute airway pressure rather than the ventilation mode—has not been tested with a quantitative endpoint.

We therefore conducted a randomised controlled trial in adults undergoing general anaesthesia with the i-gel, without a gastric drain tube, to test whether PCV reduces gastric insufflation compared with VCV, with the postoperative gastric antral cross-sectional area measured by ultrasonography as the primary outcome. We hypothesised that PCV, by lowering peak inspiratory pressure, would reduce the postoperative gastric antral cross-sectional area compared with VCV.

## 2. Materials and Methods

### 2.1. Study Design, Ethics, and Registration

This single-centre, prospective, parallel-group, randomised controlled trial was conducted at Severance Hospital, Yonsei University Health System (Seoul, Republic of Korea). The protocol was approved by the Institutional Review Board of Severance Hospital, Yonsei University Health System (approval no. 4-2015-0280; 26 May 2015) and registered at ClinicalTrials.gov on 3 June 2015, before participant enrolment (NCT02466295). Written informed consent was obtained from all participants. The oropharyngeal leak pressure measurement and the fibreoptic laryngeal view were protocol-specified research assessments included in the approved protocol and the informed-consent document. Recruitment and data collection took place from June 2015 to January 2016. The study is reported in accordance with the Consolidated Standards of Reporting Trials (CONSORT) 2025 statement [10] and its extension for non-pharmacological treatments; the completed checklist is provided as Table S1. Patients and the public were not involved in the design, conduct, or reporting of this trial.

### 2.2. Participants

Adults aged 20 years or older scheduled for elective surgery under general anaesthesia with a supraglottic airway were eligible. Exclusion criteria were: upper gastrointestinal or open abdominal surgery; absence of preoperative fasting; anticipated surgical duration of 4 h or more; anticipated difficult intubation (a history of difficult intubation, modified Mallampati class 3 or 4, or an inter-incisor distance < 3 cm at maximal mouth opening); body mass index > 35 kg·m^−2^; and pregnancy. The type of surgery was not restricted a priori; because a supraglottic airway is routinely used for breast surgery at our institution, all enrolled participants were women undergoing elective breast surgery.

### 2.3. Randomisation, Allocation Concealment, and Blinding

Participants were randomly assigned in a 1:1 ratio to the PCV or VCV group using a computer-generated randomisation sequence (https://www.randomizer.org). Allocation was concealed using sequentially numbered, opaque, sealed envelopes, which were opened only upon the participant’s arrival in the operating room.

Owing to the nature of the intervention, the attending anaesthesiologist who set the ventilator could not be blinded to group allocation. To protect the integrity of the primary outcome, gastric ultrasound images were acquired and measured by two different investigators, and the investigator who measured and analysed the gastric antral images was blinded to group allocation.

### 2.4. Anaesthesia and Airway Management

No premedication was given. After standard monitoring, general anaesthesia was induced with propofol and either remifentanil or fentanyl, followed by rocuronium 0.3 mg·kg^−1^ (approximately one ED_95_) to facilitate i-gel insertion, a recognised use of low-dose rocuronium that improves insertion conditions and sealing [11]; no maintenance dose was given. Mask ventilation was provided with 100% oxygen and either sevoflurane or desflurane using volume-controlled mask ventilation (tidal volume 8 mL·kg^−1^ of actual body weight, fresh gas flow 2 L·min^−1^, respiratory rate 15 min^−1^) for approximately 2.5–3 min, until adequate relaxation for insertion was confirmed clinically by the absence of a response to jaw thrust. The choice of opioid and volatile anaesthetic was left to the attending anaesthesiologist’s discretion, and the agents and doses were not recorded as trial variables.

A lubricated i-gel (Intersurgical Ltd., Wokingham, UK) was then inserted in the head-extended position by an anaesthesiologist experienced in its use. Size was selected according to the manufacturer’s body-weight recommendation (size 3, 30–60 kg; size 4, 50–90 kg; size 5, 90 kg or more). Correct placement was confirmed by a square-wave capnography trace during ventilation. When ventilation was inadequate, the position was optimised (head extension, jaw thrust, neck flexion, or depth adjustment); if ventilation remained inadequate, the device was removed and reinserted, up to a total of three attempts. The number of insertion attempts per participant was not systematically recorded; no participant was withdrawn for insertion failure or required tracheal intubation. After three failed attempts, the airway was secured with a tracheal tube and the participant was withdrawn, with no further data collected. No gastric drain tube was inserted through the gastric channel of the i-gel at any time during the study, so that any gastric insufflation would not be vented and could be detected on ultrasonography. Neuromuscular function was not monitored during the trial; at the end of anaesthesia, neuromuscular blockade was reversed as clinically indicated, and the agent used was not recorded.

### 2.5. Ventilation Protocol (Intervention)

After successful i-gel placement, ventilation was delivered according to group allocation using a Dräger Primus anaesthesia workstation (Dräger, Lübeck, Germany). The PCV group received pressure-controlled ventilation with the inspiratory pressure set after i-gel placement to achieve a tidal volume of 8 mL·kg^−1^ of actual body weight, which was not systematically re-titrated during surgery; the VCV group received volume-controlled ventilation with a set tidal volume of 8 mL·kg^−1^ of actual body weight. No positive end-expiratory pressure was applied (zero end-expiratory pressure) in either group. In both groups, fresh gas flow was 2 L·min^−1^, the maintenance fraction of inspired oxygen was 0.5, and respiratory rate was 8–15 min^−1^, adjusted to maintain an end-tidal carbon dioxide of 35–40 mmHg. The inspiratory-to-expiratory ratio, inspiratory rise time, flow pattern, and inspiratory pause were left at the ventilator’s default settings and were therefore consistent across patients within each ventilation mode, although the individual values were not recorded. Ventilation was fully controlled, with no assist or backup mode enabled, and apnoea was maintained throughout; no participant made spontaneous respiratory effort or triggered the ventilator. After the allocated ventilation had been established, the position of the laryngeal inlet was graded by fibreoptic bronchoscopy through the device, to record laryngeal alignment rather than to confirm ventilation, on a four-point score adapted from Brimacombe and Berry [12], numbered here in ascending order of laryngeal obstruction (grade 1, vocal cords only; grade 2, vocal cords and part of the epiglottis; grade 3, epiglottis only; grade 4, neither vocal cords nor epiglottis visible). The bronchoscope was removed before the oropharyngeal leak pressure was measured.

### 2.6. Measurements

Oropharyngeal leak pressure. With the adjustable pressure-limiting valve closed and a fixed fresh gas flow of 3 L·min^−1^, the airway pressure at which an audible leak occurred at the mouth was recorded as the oropharyngeal leak pressure (measured once, after i-gel placement), with a maximum allowed airway pressure of 40 cmH_2_O.

Ventilatory variables. Peak inspiratory pressure and expiratory tidal volume were recorded at two time points—before the start of surgery and immediately after the end of surgery—each as the mean of three readings.

Gastric antral ultrasonography. Gastric ultrasound of the antrum was performed after i-gel placement and establishment of the allocated ventilation but before the start of surgery, and immediately after the end of surgery (before i-gel removal and before emergence). In the sagittal plane at the level of the gastric antrum, two perpendicular diameters—craniocaudal (CC) and anteroposterior (AP)—of a single antral cross-section were measured, and the antral cross-sectional area was calculated as area = (π × CC × AP)/4 [4]. Three images were obtained at each time point and averaged.

Adverse events. Oxygen desaturation (peripheral oxygen saturation < 93%, a conservative threshold, stricter than the conventional < 90%), laryngospasm, and regurgitation were monitored throughout anaesthesia and emergence. Regurgitation was assessed by inspection of the i-gel and oropharynx for gastric content at device removal, and aspiration by oxygen saturation, peak airway pressure, and the capnograph trace; no dye or pH testing was used.

### 2.7. Outcomes

The primary outcome was the gastric antral cross-sectional area measured after the end of surgery. Secondary outcomes were peak inspiratory pressure, oropharyngeal leak pressure, and expiratory tidal volume. In a post hoc, exploratory analysis, gastric insufflation was classified as a >30% increase in antral cross-sectional area from baseline, following a definition adopted in subsequent gastric ultrasonography studies [13,14]. The antral cross-sectional area is an indirect measure that reflects total antral content rather than direct detection of gas entry, and this >30% threshold has not been validated for prolonged ventilation through a supraglottic airway; the insufflation outcome is therefore regarded as exploratory. Throughout, we distinguish the measured antral cross-sectional area from gastric insufflation, which is inferred from it.

### 2.8. Sample Size

The sample size was based on an internal pilot study (IRB no. 4-2015-0110), in which the postoperative antral cross-sectional area was 524 ± 119 mm^2^ with VCV and 400 ± 93 mm^2^ with PCV. To detect a between-group difference of 100 mm^2^ in postoperative antral cross-sectional area, assuming a standard deviation of 119 mm^2^, a two-sided α of 0.05, and a power of 90% (Student’s *t*-test), 31 participants per group were required. Allowing for a 10% dropout rate, 34 participants per group (68 in total) were enrolled. No interim analysis was performed. The pilot study has not been published, and no pilot participant was included in the present trial.

### 2.9. Statistical Analysis

The distribution of continuous variables was assessed with the Shapiro–Wilk test. The pre-specified primary analysis, as registered, was the comparison of the postoperative antral cross-sectional area between the two groups using Student’s *t*-test. Because the Shapiro–Wilk test indicated departures from normality, the Mann–Whitney U test and a baseline-adjusted analysis of covariance (ANCOVA) are additionally reported as supportive analyses; the three analyses are interpreted together. Continuous variables are presented as mean ± standard deviation or median [interquartile range]; between-group differences are reported as differences in means (PCV − VCV) with 95% confidence intervals, and secondary continuous outcomes were compared using Student’s *t*-test with Welch’s correction for unequal variances. Categorical variables are presented as numbers (percentages) and were compared using Fisher’s exact test. Between-group differences are expressed as PCV − VCV with 95% confidence intervals. All participants were analysed as randomised, with no missing data. In a post hoc sensitivity analysis, the baseline-adjusted ANCOVA was repeated with anaesthetic time added as a covariate, and the association between the change in antral cross-sectional area and anaesthetic and operation time was assessed using Spearman’s rank correlation. Secondary and exploratory comparisons were not adjusted for multiplicity and are interpreted as hypothesis-generating. A two-sided *p*-value < 0.05 was considered statistically significant. Analyses were performed using Python 3.9 (SciPy 1.13, statsmodels 0.14, NumPy 2.0, and pandas 2.3). The statistical analysis code (Python) was prepared with the assistance of a generative AI tool (Claude Opus 4.8, Anthropic, San Francisco, CA, USA); all code, outputs, and results were reviewed, validated, and verified by the authors. The study design, data collection, analyses, and interpretation are the authors’ own.

## 3. Results

### 3.1. Participant Flow and Baseline Characteristics

**Figure 1:**
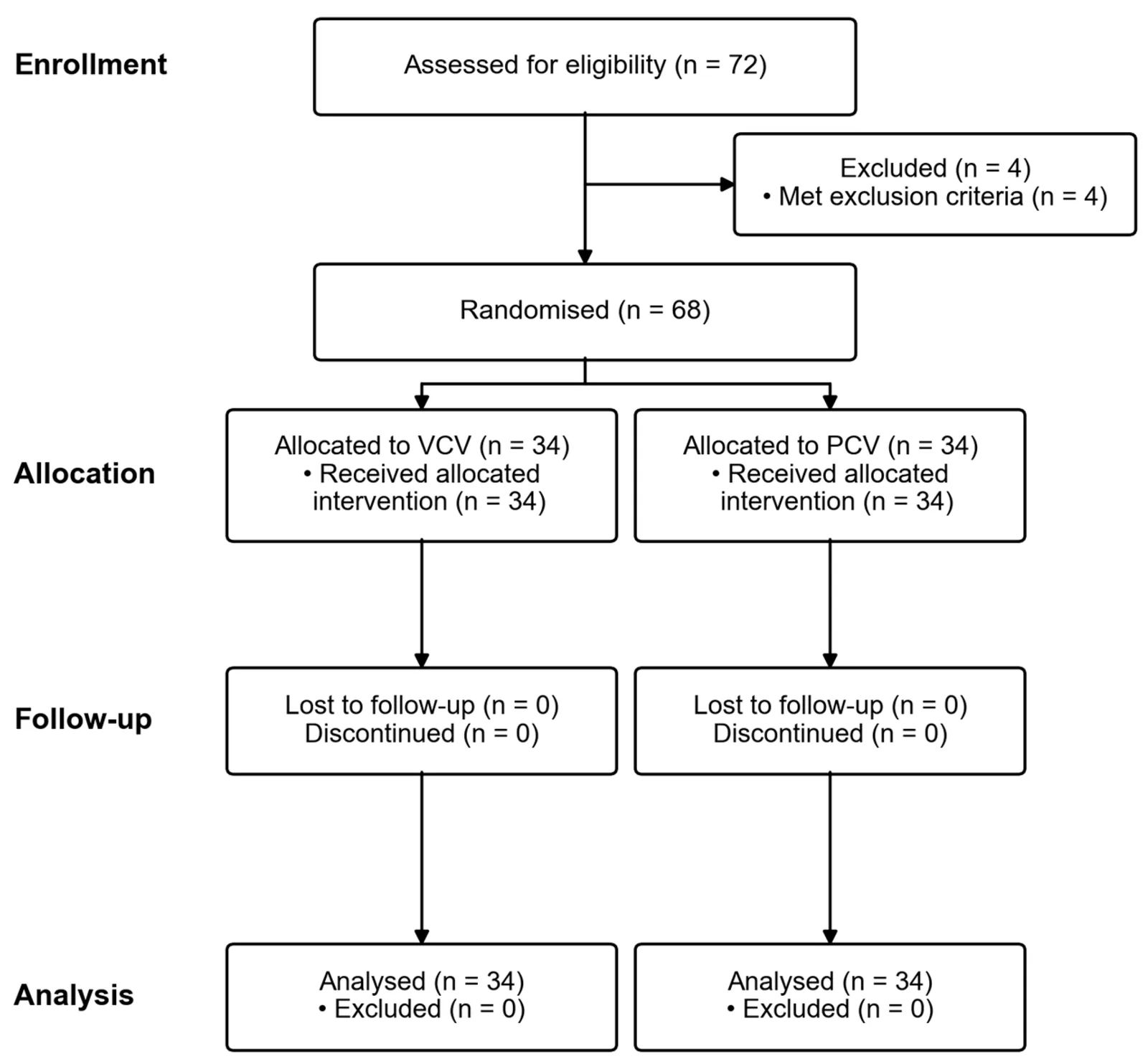
CONSORT flow diagram of participant enrolment, allocation, follow-up, and analysis. PCV, pressure-controlled ventilation; VCV, volume-controlled ventilation.

Of 72 patients assessed for eligibility, 4 were excluded because they met exclusion criteria, and 68 were randomised (34 to PCV and 34 to VCV). All 68 randomised participants completed the study and were analysed; there was no loss to follow-up (Figure 1). All participants were women undergoing elective breast surgery.

Baseline demographic and clinical characteristics, including age, height, weight, body mass index, ASA physical status, i-gel size, fibreoptic laryngeal view grade, operation and anaesthetic times, and the baseline gastric antral cross-sectional area, are shown in Table 1.

### 3.2. Primary Outcome

**Figure 2:**
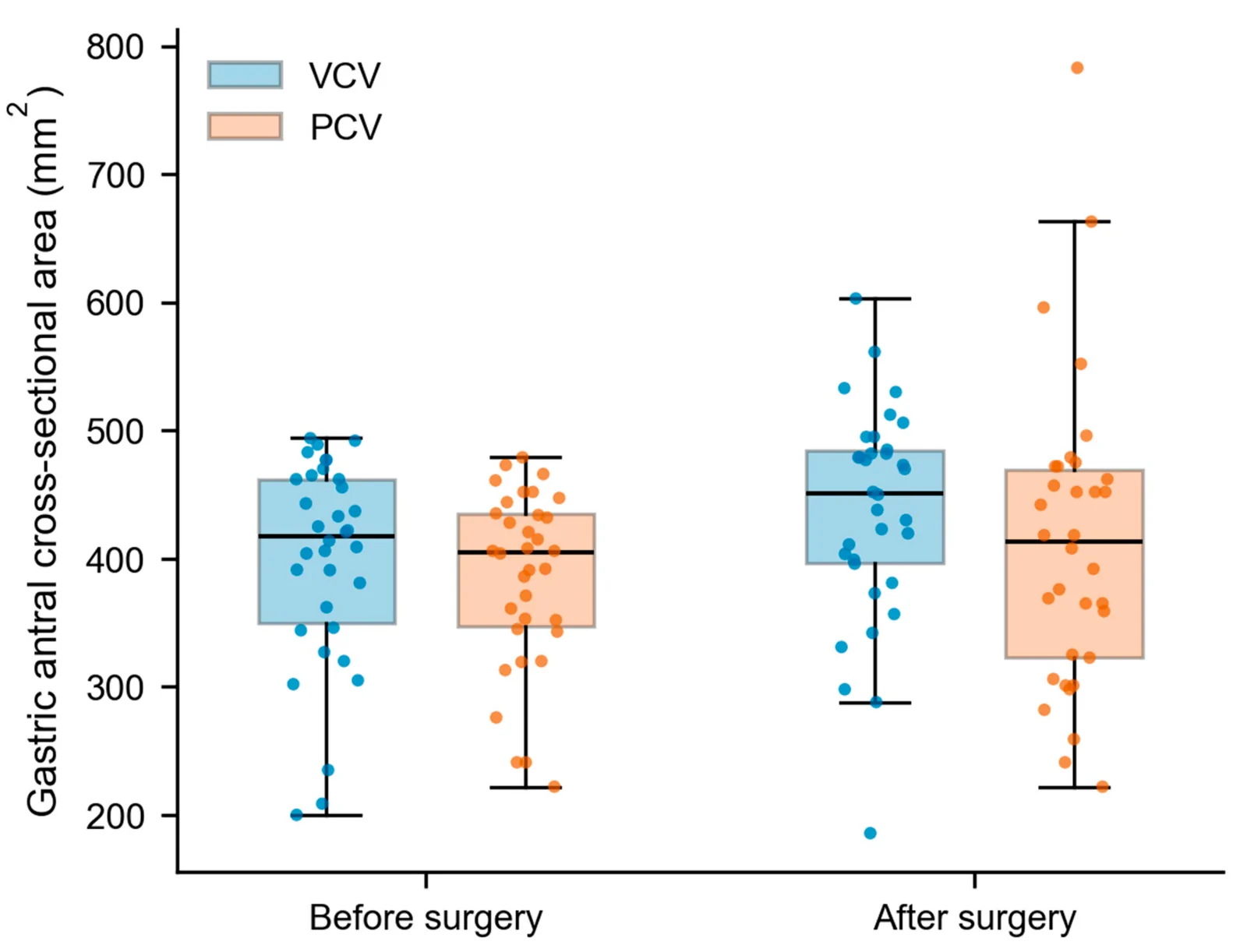
Gastric antral cross-sectional area before and after surgery in the volume-controlled (VCV) and pressure-controlled (PCV) ventilation groups. Boxes denote the median and interquartile range, whiskers extend to the most extreme values within 1.5 × the interquartile range, and dots represent individual participants. The postoperative antral cross-sectional area did not differ significantly between groups (*p* = 0.094). PCV, pressure-controlled ventilation; VCV, volume-controlled ventilation.

The postoperative gastric antral cross-sectional area did not differ between groups. In the pre-specified primary analysis (Student’s *t*-test), the mean difference (PCV − VCV) was −23.7 mm^2^ (95% CI −73.9 to 26.4; *p* = 0.347) (Table 2, Figure 2). This result was consistent in the supportive analyses: the Mann–Whitney U test (*p* = 0.094) and the baseline-adjusted analysis of covariance (adjusted difference −9.6 mm^2^; 95% CI −43.1 to 23.9; *p* = 0.568). Both confidence intervals exclude a difference of 100 mm^2^, the effect the trial was powered to detect; the data are therefore incompatible with a between-group difference of that magnitude, while remaining compatible with smaller differences in either direction. In a sensitivity analysis adding anaesthetic time to the baseline-adjusted model, the result was unchanged (adjusted difference −10.3 mm^2^; 95% CI −44.1 to 23.5; *p* = 0.55).

### 3.3. Secondary and Exploratory Outcomes

The incidence of gastric insufflation, defined post hoc as a >30% increase in antral area, did not differ between groups (2/34 with VCV vs. 3/34 with PCV; *p* = 1.000; Table 2). All five participants who met this threshold had a fibreoptic view of grade 2; the three participants with a grade-3 view (all in the VCV group) did not insufflate. Insufflation therefore did not cluster in malpositioned devices. Change in antral area was not correlated with anaesthetic or operation time (Spearman ρ ≈ 0.03). Operation and anaesthetic times ranged from 16 to 139 min and from 35 to 165 min, respectively.

Both before and after surgery, peak inspiratory pressure and expiratory tidal volume were lower in the PCV group than in the VCV group (*p* ≤ 0.002), whereas the oropharyngeal leak pressure was similar between groups (Table 2). In every patient, the peak inspiratory pressure remained below the oropharyngeal leak pressure, and no audible leak was detected. Because respiratory rate was titrated to a target end-tidal carbon dioxide of 35–40 mmHg, ventilation was managed to a normocapnia target in both groups; individual end-tidal carbon dioxide values were not recorded.

### 3.4. Adverse Events

No oxygen desaturation, laryngospasm, regurgitation, aspiration, or other adverse event occurred in either group, and no participant required conversion to tracheal intubation after randomisation.

## 4. Discussion

In adults ventilated through an i-gel without a gastric tube, and within the low airway pressures that this device produced, pressure-controlled ventilation gave a lower peak inspiratory pressure than volume-controlled ventilation, but the postoperative antral cross-sectional area and the exploratory incidence of gastric insufflation did not differ detectably between the modes. We interpret this as indicating that, in this setting, the ventilation mode was not a detectable determinant of gastric insufflation—not that the two modes are equivalent, which this trial was neither designed nor powered to demonstrate. Both the unadjusted and baseline-adjusted confidence intervals excluded the effect the trial was powered to detect, so the data are incompatible with a difference of that magnitude while remaining compatible with smaller differences in either direction.

Because PCV lowers peak pressure, it is often regarded as the gentler mode for the stomach and has been reported to ventilate children more efficiently than VCV with a laryngeal mask [7]. Our data did not support this assumption in adults when the antrum was assessed by ultrasonography [4,5]. The antral cross-sectional area is, however, an indirect measure that reflects total antral content rather than direct detection of gas entry, and should be interpreted as such.

The low incidence of insufflation is consistent with the low pressures used. During facemask ventilation, ultrasonographically detected insufflation rises steeply as inspiratory pressure approaches ~20 cmH_2_O [5,13,15]. In our trial, peak pressure was well below this: the median was about 9 cmH_2_O, roughly 15 cmH_2_O below the oropharyngeal leak pressure (~24–25 cmH_2_O), and in no patient did peak pressure reach the leak pressure. Facemask and supraglottic ventilation differ in where the seal lies and how pressure is transmitted to the oesophagus, so this dose–response relationship is offered only as an illustrative frame, not as direct evidence. Within this low-pressure range, the ~2 cmH_2_O that separated the two modes was too small to produce a detectable difference in insufflation.

Low pressure did not abolish insufflation: five participants (7%) reached the threshold, four of them at low peak pressure (7–12 cmH_2_O). Several non-exclusive factors may contribute. The oropharyngeal leak pressure reflects the oral (peri-laryngeal) seal rather than the oesophageal opening pressure, which was not measured and which is reduced under general anaesthesia; small per-breath volumes may accumulate over an operation; and, because insufflation was defined as a percentage increase, the threshold is crossed more readily when the baseline antrum is small. All five events occurred with a grade-2 fibreoptic view, so device malposition did not account for them. Based on only five events split across both groups, these observations are hypothesis-generating.

The result is consistent with the paediatric i-gel comparison, in which PCV lowered peak pressure without reducing insufflation [8]; that study enrolled children aged 6–84 months, in whom insufflation was more frequent (~21%) than in our adults (7%). That a similar result emerged in dissimilar populations suggests the finding is not incidental. It also aligns with adult work implicating airway pressure rather than mode: insufflation has not been detected with the i-gel during pressure-controlled ventilation at moderate pressures [16], and antral area does not differ among supraglottic devices [9].

Two cautions apply. First, this does not mean airway pressure is unimportant; a supraglottic airway does not separate the airway from the oesophagus, and keeping airway pressure low remains the relevant safeguard. However, the oropharyngeal leak pressure tests the oral seal, not the gastro-oesophageal barrier, so the margin between peak pressure and leak pressure should not be read as a direct measure of protection against insufflation. Second, the lower delivered tidal volume in the PCV group is intrinsic to a pressure-targeted mode, in which delivered volume is the dependent variable; because the peak pressure difference was accompanied by a tidal-volume difference, we report peak pressure descriptively and do not claim it as a benefit of PCV. Ventilation was managed to a normocapnia target in both groups, although individual end-tidal carbon dioxide values were not recorded.

Within the low-pressure range studied, and in a population of this kind, the choice between pressure- and volume-controlled ventilation need not be driven by concern for gastric insufflation; it can rest on other considerations such as lung mechanics or clinician familiarity. Attention is better directed at confirming correct device placement and an adequate seal.

This study has several limitations. It was a single-centre trial in a homogeneous cohort—women undergoing breast surgery, ASA physical status I–II, with obesity (body mass index > 35) excluded—so the findings do not extend to men, other procedures, or obese patients, in whom gastric insufflation carries the greatest consequence. The primary outcome is an indirect measure: a single supine antral cross-section does not capture gas in the gastric body or fundus, and antral area may also change with gastric secretion, redistribution of contents, gastric emptying, or measurement variability; averaging three readings at each time point reduced but did not eliminate this. Although antral cross-sectional area ultrasonography is an established, validated technique [4,5] and the measurements were made by a single blinded investigator, we did not prospectively assess intra- or inter-observer reliability in this cohort. The insufflation endpoint (a >30% increase in antral area) was defined post hoc, has not been validated for prolonged supraglottic ventilation, and rested on only five events, so it is exploratory. The two modes were applied pragmatically and were not matched for delivered tidal volume, which qualifies the peak pressure comparison. Peak inspiratory pressure remained far below the oropharyngeal leak pressure in every patient, creating a floor effect; the trial therefore does not address higher airway pressures or reduced pulmonary compliance, the situations of greatest clinical concern. Neuromuscular function was not monitored and a single insertion dose of rocuronium was used, so block is likely to have waned during surgery; any contribution of neuromuscular blockade to the reduced oesophageal barrier therefore applies chiefly to the early ventilation period, and the findings apply to i-gel ventilation initiated under neuromuscular blockade and cannot be extrapolated to the unparalysed case. End-tidal carbon dioxide, inspired oxygen fraction, and opioid and volatile agent exposure were not recorded as trial variables, so ventilatory equivalence between groups cannot be demonstrated numerically. Oropharyngeal leak pressure was measured once, early, and may not represent the seal throughout surgery, so end-of-surgery re-measurement would be valuable in future work; oesophageal opening pressure was not measured. No case exceeded three hours, so the trial does not address prolonged cases, in which seal degradation and cumulative insufflation may matter more. The sample size was based on an unpublished internal pilot whose effect estimate (124 mm^2^) exceeded that observed (~24 mm^2^) and is likely to have been inflated; the trial was powered for the primary continuous outcome and not for the binary insufflation endpoint. Finally, analysis and reporting were delayed by clinical service demands during the COVID-19 pandemic (2020–2022) and subsequent disruptions to the Korean medical workforce (2024); neither the i-gel device nor the ventilation modes have changed materially in the interval, so the findings remain current. Tidal volume was set on actual body weight; in this normal-weight cohort, this differs little from predicted body weight, but the basis should be considered when generalising to patients with obesity. The i-gel was used deliberately without a gastric drain tube—necessary to detect insufflation but a departure from routine second-generation use—so the findings apply to i-gel use without gastric venting.

## 5. Conclusions

In adults ventilated through an i-gel within a low airway-pressure range, pressure-controlled ventilation lowered peak inspiratory pressure but did not reduce the postoperative antral cross-sectional area or the exploratory incidence of gastric insufflation compared with volume-controlled ventilation. These findings apply to women undergoing breast surgery (ASA I–II, body mass index ≤ 35) ventilated with neuromuscular blockade in this low airway-pressure range; they do not establish equivalence between the modes and do not extend to higher airway pressures, reduced compliance, or obesity. Within these limits, the choice between pressure- and volume-controlled ventilation need not be governed by concern for gastric insufflation.

## Figures and Tables

**Table 1:** Baseline characteristics.

Characteristic	VCV (*n* = 34)	PCV (*n* = 34)
Age, yr	45.0 ± 10.0	46.3 ± 11.8
Height, cm	158.8 ± 5.3	160.1 ± 4.9
Weight, kg	55.7 ± 6.6	57.3 ± 7.6
BMI, kg/m^2^	22.2 [20.7–23.6]	21.7 [20.9–23.1]
ASA I/II, n (%)	33 (97.1)/1 (2.9)	32 (94.1)/2 (5.9)
i-gel size 3/4, n (%)	28 (82.4)/6 (17.6)	25 (73.5)/9 (26.5)
Fibreoptic view grade 1/2/3/4, n	2 (5.9)/29 (85.3)/3 (8.8)/0 (0)	4 (11.8)/30 (88.2)/0 (0)/0 (0)
Operation time, min	44.5 [27.0–62.2]	54.5 [35.2–82.8]
Anaesthetic time, min	70.0 [55.0–98.8]	75.0 [58.5–112.5]
Baseline antral CSA, mm^2^	417.4 [350.0–462.0]	405.0 [346.8–435.0]

All participants were women undergoing elective breast surgery. Values are mean ± SD, median [IQR], or n (%). No between-group significance tests were performed on baseline variables, in accordance with CONSORT. ASA, American Society of Anesthesiologists physical status; BMI, body mass index; CSA, cross-sectional area; IQR, interquartile range; PCV, pressure-controlled ventilation; SD, standard deviation; VCV, volume-controlled ventilation.

**Table 2:** Ventilatory variables and gastric insufflation.

Outcome	VCV (*n* = 34)	PCV (*n* = 34)	Difference (95% CI)	*p*
Primary outcome				
Postoperative antral CSA, mm^2^	451.0 [396.8–484.2]	413.1 [323.3–469.5]	−23.7 (−73.9, 26.4) a	0.347
Baseline-adjusted (ANCOVA)	—	—	−9.6 (−43.1, 23.9)	0.568
Secondary and exploratory outcomes				
Gastric insufflation, n (%)	2 (5.9)	3 (8.8)	+2.9% b	1.000
Peak inspiratory pressure, cmH_2_O				
Before surgery	10.0 [9.0–11.8]	8.0 [7.0–9.0]	−1.9 (−2.8, −1.0)	<0.001
After surgery	10.0 [9.0–12.0]	8.5 [8.0–10.0]	−1.7 (−2.6, −0.8)	<0.001
Oropharyngeal leak pressure, cmH_2_O	24.4 ± 4.0	23.7 ± 3.2	−0.6 (−2.4, 1.1)	0.461
Expiratory tidal volume, mL				
Before surgery	464.5 [433.8–479.5]	408.5 [374.2–453.8]	−47.2 (−76.6, −17.9)	0.002
After surgery	459.0 [419.5–490.5]	383.5 [361.2–429.8]	−57.4 (−86.8, −28.1)	<0.001

Values are mean ± SD or median [IQR]. Between-group differences are differences in means (PCV − VCV) with 95% CI; the primary outcome used the pre-specified Student’s *t*-test and other continuous outcomes Welch’s *t*-test, and the baseline-adjusted ANCOVA and the Mann–Whitney U test (*p* = 0.094) are shown as supportive analyses of the primary outcome. CSA, cross-sectional area; CI, confidence interval; PCV, pressure-controlled ventilation; VCV, volume-controlled ventilation. a Mean difference (PCV − VCV); group values are summarised as median [IQR]. b Risk difference (PCV − VCV).

## Data Availability

The data presented in this study are available from the corresponding author upon reasonable request; they are not publicly available due to privacy restrictions.

## Supplementary Materials

The following supporting information can be downloaded at: https://www.mdpi.com/article/10.3390/medicina62091657/s1, Table S1: CONSORT 2025 checklist of information to include when reporting a randomised trial *.

## Abbreviations

The following abbreviations are used in this manuscript:

ANCOVA	Analysis of covariance
AP	Anteroposterior
ASA	American Society of Anesthesiologists
BMI	Body mass index
CC	Craniocaudal
CI	Confidence interval
CSA	Cross-sectional area
IQR	Interquartile range
PCV	Pressure-controlled ventilation
SD	Standard deviation
VCV	Volume-controlled ventilation
